# LGB prevalence in schools is associated with unhealthy weight-control behaviors in lesbian, gay, and bisexual youth: a multilevel analysis

**DOI:** 10.1186/s12889-021-11260-3

**Published:** 2021-06-29

**Authors:** Carolina da Franca Bandeira Ferreira Santos, Fabiana Godoy, Valdenice Aparecida de Menezes, Viviane Colares, Patrícia Maria Pereira de Araújo Zarzar, Raquel C. Ferreira, Ichiro Kawachi

**Affiliations:** 1 Graduate Program in Hebiatrics - School of Dentistry, University of Pernambuco, Av. Gov. Agamenon Magalhães - Santo Amaro, Recife, PE 50100-010 Brazil; 2 Pediatric Dentistry and Orthodontics, School of Dentistry, Federal University of Minas Gerais, R. Prof. Moacir Gomes de Freitas, 688 - Pampulha, Belo Horizonte, MG 31270-901 Brazil; 3 Social and Preventive Dentistry, School of Dentistry, Federal University of Minas Gerais, R. Prof. Moacir Gomes de Freitas, 688 - Pampulha, Belo Horizonte, MG 31270-901 Brazil; 4 Social and Behavioral Sciences, Harvard T.H. Chan School of Public Health, 677 Huntington Ave, Boston, MA 02115 USA

**Keywords:** Sexual and gender minorities, Schools, Feeding and eating disorders, Adolescent, Multilevel analysis

## Abstract

**Background:**

Previous studies have found that a school climate of more heteronormativity is associated with adverse effects on the mental health of LGB students. Accordingly, our aim was to assess the association between lower LGB prevalence in schools and unhealthy weight-control behaviors among LGB youth.

**Methods:**

A cross-sectional, multilevel study based in public high schools in the city of Olinda, Northeast Brazil. A multilevel logistic regression was performed, including 2500 adolescents enrolled in 27 schools. The contextual variable was the prevalence of LGB youth in each school (as a proxy for heteronormativity in schools), while the outcome was unhealthy weight-control behaviors (fasting, purging, and taking diet pills). We controlled for socioeconomic characteristics (age, sex, receiving a family allowance), obesity, and self-reported happiness.

**Results:**

Lower LGB prevalence in schools was associated with higher odds of engaging in unhealthy weight-control behaviors (OR: 1.5, 95%CI: 1.0, 2.2) among all youth, regardless of sexual orientation. No cross-level interactions between school context and individual characteristics were statistically significant.

**Conclusion:**

Lower LGB prevalence in schools was associated with a higher risk of unhealthy weight-control behaviors in youth regardless of sexual orientation, which may reflect either the contextual influence of school climate, or may be due to residual confounding.

## Background

Schools need to provide a safe environment to guarantee the healthy development of their students. However, these conditions are not always met for sexual and gender minority youth as they are more likely to report negative perceptions and experiences of their school environment [1, 2]. Moreover, schools with more heteronormative norms have been shown to be associated with increased likelihood of depressed mood for all boys, regardless of their level of gender typicality [3]. Gower et al. [4] found that schools with more supportive LGBT climates had students reporting lower odds of victimization, regardless of their sexual orientation. Poteat et al. [5] showed that homophobic victimization had a direct effect on suicide behavior and school belonging, and an indirect effect on educational outcomes (reported grades, truancy, and graduation) for all students, regardless of sexuality. A Brazilian study which examined discrimination against homosexuality found that students who reported experiencing homophobic discrimination evaluated their school experience less positively [6]. In sum, heteronormativity in school is harmful to all students, not just to sexual and gender minorities. According to Johns et al. [7], there is a need for more school-based research focusing on the health of sexual and gender minority youth utilizing a multi-level framework to consider the influence of school contexts and climate. In the present study, we examined whether lower LGB prevalence (as a measure of school context) is associated with unhealthy weight-control behaviors among youth, irrespective of their sexual orientation.

Outwardly, Brazil projects an image of a liberal-progressive country with regard to issues of sexuality, probably because of its famous Carnival and the visibility of gay activists [8]. Contrary to that popular image, however, Brazil is one of the countries with the highest toll of deaths attributed to homophobic violence [9], and the numbers in the Northeast of Brazil are among the worst [10]. Despite advances in LGBT rights during the last few years [11], it is important to consider that virtually all the progress in Brazilian LGBT legislation was based on judicial fiat instead of legislation proposed at the federal level [8]. In addition, in the wake of the last presidential election, the Brazilian population is becoming more intolerant. The Inter-American Commission on Human Rights has declared that the life of a gay member of the Brazilian Congress was “at grave risk” and that the state was not doing enough to protect them. Fear has driven some politicians to voluntarily drive themselves into exile [12]. The Brazilian president and his staff have repeatedly made overt homophobic statements in public [13, 14]. This homophobic tendency is already reflected in policies such as the removal of sex education from the Brazilian Education Plan [15] and a bill proposed by a senator for criminalizing homosexuality in schools [16].

### Minority stress and health behaviors

Brazilian adolescents report widespread negative and even violent reactions from their families when they “came out” to reveal their sexual orientation [17]. Minority stress refers to the psychological trauma associated with widespread societal prejudice, i.e. against sexual minorities [18]. Some Brazilian studies have evaluated minority stress. One study showed that minority stress predicted depressive symptomatology among sexual minority men [19], while another study demonstrated that the odds ratio for suicide attempts more than doubled among young Brazilians experiencing sexual stigma [20].

One mental health consequence of minority stress manifests in the form of eating disorders. Previous studies have demonstrated a higher prevalence of these disorders among sexual and gender minorities [21, 22]. According to Mensinger et al. [23], greater experiences of bullying/abuse are more prevalent in sexual and gender minorities and probably explain more severe eating disorders symptoms in this group compared to cisgender heterosexuals.

To our knowledge, the school climate regarding hostility was not studied relating to sexual minorities and weight control behaviors. We hypothesized that strongly heteronormative school contexts (i.e. lower prevalence/visibility of sexual minority youth) are associated with more unhealthy weight control behaviors for all students, regardless of their sexual orientation. For heterosexual youth, we hypothesize that masculine and feminine body ideals transmitted by society (i.e. through advertising and social media) are more likely to prevail in strongly heteronormative school contexts, and that pressure to conform to these ideals will result in higher prevalence of unhealthy weight control behaviors.

For sexual minority youth, we hypothesize that more strongly heteronormative school contexts lead to more discrimination against sexual minorities, resulting in higher minority stress, and unhealthy coping responses (in the form of unhealthy weight control behaviors). Hence, our hypothesis is that strongly heteronormative schools will result in unhealthy weight control behaviors for both heterosexual youth and sexual minority youth.

In the present study we sought to examine the association between heteronormativity (as proxied by the prevalence of LGB youth in schools) and unhealthy weight-control behaviors among sexual minority youth.

## Methods

This is a cross-sectional study performed in Olinda in the state of Pernambuco, Brazil. Olinda had a population of 390,000 residents and 33 state public high schools in 2018.

The data were collected from February to June 2018 in 87% (*n* = 27) of all state public high schools with daytime classes (31 schools), which agreed to take part in the study. The two schools offering only night classes were not invited. The response rate of all invited students enrolled in the 27 schools was 37.2% (2700). Almost all the classrooms (91%) in the involved schools participated in our data collection.

The Informed Consent Form (ICF) was signed by the parents/guardians of all participants under 18-years-old, while children also provided written assent. Participants over 18-years of age consented by themselves. This study was approved by the Ethics Committee of the University of Pernambuco (No. 2.361.780).

Four trained and calibrated examiners measured body weight and height with strong inter-rater reliability for height (kappa 0.99). A portable stadiometer (Sanny®) and a self-zeroing digital scale (CAMRY) with a maximum capacity of 150 kg were used to measure height and body weight, respectively. The participants were weighed barefoot wearing socks and light clothing.

The sociodemographic and health behavior modules were adapted from the U.S. Youth Risk Behavior Survey (YRBS) questionnaire [24]. The student’s sociodemographic situation was assessed by their age, sex, and whether they received a family allowance. The family allowance is given in Brazil to poor families and extremely poor families with per-capita monthly incomes below $170 BRL (~$45 USD) or $85 BRL (~$23 USD), respectively.

The survey also asked whether the students had experienced victimization, whether they were actively trying to lose weight, as well as self-perception of happiness (In general, how do you feel in your actual life?) on a 5-point Likert scale (1 “totally unhappy” 2 “not happy” 3 “happy” 4 “very happy” 5 “totally happy”).

Victimization was based on at least one positive answer for these three questions: (1. During the past 12 months, have you ever been bullied on school property? - answer choices: 1 “yes” 2 “no”; 2. During the past 12 months, have you ever been electronically bullied? (Count being bullied through texting, Instagram, Facebook, WhatsApp or other social media.) - answer choices: 1 “yes” 2 “no”; 3. During the past 12 months, how many times has someone threatened or injured you with a weapon such as a gun, knife, or club on school property?) - answer choices: 1 “0 times” 2 “1 time” 3 “2 or 3 times” 4 “4 or 5 times” 5 “6 or 7 times” 6 “8 or 9 times” 7 “10 or 11 times” 8 “12 or more times”. Trying to lose weight was based in the question “Which of the following are you trying to do about your weight?”, with responses categorized as: trying to gain weight, stay the same weight or I am not trying to do anything about my weight, and trying to lose weight. Weight categories were based on cut-off z scores recommended by the WHO: underweight (< − 2), normal weight (≥ − 2 and < + 1), overweight (≥ + 1 and < + 2), obese (≥ + 2) [25].

The LGB variable was derived from adolescents who reported having sexual intercourse with a partner of the same sex or with partners of both sexes in line with Kim et al. [26], and Parkes et al. [27]

Our contextual variable of interest was LGB prevalence in each school as a proxy of LGB visibility in line with VanKim et al. [28]. The variable was dichotomized into lower LGB prevalence (≤ 3.01%) vs middle-lower, middle-higher, and higher LGB prevalence (> 3.01%), where the cut-off was made at the 25th percentile.

The outcome variable was unhealthy weight-control behaviors created by combining the following three behaviors: fasting, purging, and using diet pills.

Data analysis was carried out using STATA/IC version 15.1 software program. We performed a sequence of multilevel logistic regressions (with random intercept) for the outcome of unhealthy weight-control behaviors (fasting, purging, or using diet pills). The empty model included only the dependent variable (Model 1). The second model included only the contextual variable (Model 2), and the third model included only individual variables. In the final model, we combined the individual and contextual variables (Model 4). The cross-level interaction between the contextual variable (LGB prevalence) and the individual variables (LGB and sex) were also tested. Some individual variables such as obesity, trying to lose weight, victimization, and self-perception of unhappiness were controlled for attenuates the association between LGB & unhealthy weight-control behaviors.

We used the proportional change in variance (PCV) to assess changes in the random intercept term. In other words, the PCV represents the proportional change in the area level variance compared to the empty model (Model 1) [29]. We also used the Median Odds Ratio (MOR) to quantify the variation between clusters in each sequence of models [30]. When the MOR equals 1.0, it means that there is no heterogeneity between the analyzed contexts. We also tested the fitted multilevel logistic regression model using the Deviance (2 Res log-likelihood).

## Results

A total of 2500 students filled out the questionnaires out of the 2700 adolescents (and parents) who consented to participate in this study (92.6% rate response). The reasons for missing data in the survey were adolescents who did not allow anthropometric measurements (*n* = 53) or did not answer most of the questionnaire (the number is specific for each variable). More participants were female (55.9%), older young (74.7%), and not receiving a family allowance (54.5%) (Table 1).

**Table 1:** Sample characteristics

Variables	N (%)
**Age**	**(N 2482)**
14-15 yrs	628 (25.3)
16–19 yrs.	1854 (74.7)
**Gender**	**(N 2438)**
Male	1076 (44.1)
Female	1362 (55.9)
**Family allowance** ^**a**^	**(N 2421)**
No	1320 (54.5)
Yes	1101 (45.5)
**LGB**^**b**^	**(N 1194)**
No	1066 (89.3)
Yes	128 (10.7)
**Victimization**^**c**^	**(N 2498)**
No	1870 (74.9)
Yes	628 (25.1)
**Self-perception of happiness**	**(N 2465)**
Fully happy/very happy/happy	2034 (82.5)
Fully unhappy/not happy	431 (17.5)
**Trying to lose weight**	**(N 2447)**
No (gain weight, stay the same weight, or doing anything about weight)	1558 (63.7)
Yes (lose weight)	889 (36.3)
**Obese**	**(N 2013)**
No (underweight, normal weight, and overweight)	1774 (88.1)
Yes	239 (11.9)
**Fasting**	**(N 2423)**
No	2097 (86.6)
Yes	326 (13.5)
**Using diet pills**	**(N 2424)**
No	2295 (94.7)
Yes	129 (5.3)
**Purging**	**(N 2412)**
No	2315 (96.0)
Yes	97 (4.0)
**Unhealthy weight-control** **(fasting, using diet pills or purging)**	**(N 2385)**
No	1957 (82.0)
Yes	428 (18.0)
**LGB prevalence in school (contextual variable)**	**(N 2500)**
Lower quartile (1.75–3.01%)	694 (27.76)
Middle-lower quartile (3.03–4.11%)	674 (26.96)
Middle-higher quartile (4.35–5.88%)	548 (21.92)
Higher quartile (6.25–14.81%)	584 (23.36)

^a^ family allowance is given in Brazil to poor families and extremely poor families with per-capita monthly incomes below $170 BRL (~$45 USD) or $85 BRL (~$23 USD), respectively. The participants answered yes (receive) or no (not receive)

^b^ LGB variable was defined from adolescents who reported having sexual intercourse with a partner of the same sex or with partners of both sexes

^c^ Victimization was based on at least one positive answer for these three questions: (1. During the past 12 months, have you ever been bullied on school property?; 2. During the past 12 months, have you ever been electronically bullied? (Count being bullied through texting, Instagram, Facebook, WhatsApp or other social media.); 3. During the past 12 months, how many times has someone threatened or injured you with a weapon such as a gun, knife, or club on school property?)

We found associations at the individual level in the expected directions, such as higher reports of unhealthy weight control behaviors among LGB youth (OR: 1.9, 95%CI, 1.1–3.2), as well as adolescents reporting victimization (OR: 1.6, 95%CI, 1.1–2.4). Over and above these individual associations, the contextual environment of lower LGB prevalence was also significantly associated with unhealthy weight-control behaviors (OR: 1.5, 95%CI, 1.0–2.2) (Table 2). However, when we tested cross-level interactions between school context and individual characteristics, they were not statistically significant (Model 5c, Table 2): LGB prevalence x LGB status (OR: 3.8, 95% CI, 0.6–23.7), LGB prevalence x sex (OR: 1.0, 95% CI, 0.4–2.2), or LGB prevalence x LGB adolescent x sex (OR: 0.0, 95% CI, 0.0–1.0). In other words, lower LGB prevalence in schools was associated with higher odds of unhealthy weight control behaviors among *both* LBG and heterosexual youth.

**Table 2:** Multilevel adjustment for LGB prevalence in school and unhealthy weight-control (fasting, using diet pills or purging)

Parameters	Empty model (Model 1)	Random intercept, fixed effects contextual variable (Model 2)	Random intercept, fixed effects individual variable (Model 3)	Random intercept, fixed effects (individual + contextual variables) (Model 4)	Random intercept, fixed effects (individual + contextual variables + Interactions) (Model 5a)	Random intercept, fixed effects (individual + contextual variables + Interactions) (Model 5b)	Random intercept, fixed effects (individual + contextual variables + Interactions) (Model 5c)
Fixed part							
Individual factors							
Constant	0.2***[0.2,0.3]	0.2***[0.2,0.3]	0.0***[0.0,0.1]	0.0***[0.0,0.1]	0.0***[0.0,0.1]	0.0***[0.0,0.1]	0.0***[0.0,0.1]
Age(16–19 vs. 14–15)			1.8[1.0,3.4]	1.8[1.0,3.4]	1.8[1.0,3.3]	1.8[1.0,3.3]	1.8[1.0,3.3]
Gender (female vs. male)			1.7**[1.2,2.4]	1.7**[1.2,2.5]	1.7*[1.2,2.5]	1.8*[1.2,2.9]	1.9*[1.1,3.1]
Family allowance (yes vs. no)			1.2[0.8,1.7]	1.2[0.8,1.7]	1.2[0.8,1.7]	1.2[0.8,1.7]	1.2[0.8,1.7]
LGB (yes vs. no)			1.7*[1.0,2.9]	1.9*[1.1,3.2]	1.8*[1.0,3.2]	1.9*[1.1,3.2]	1.9[0.8,4.6]
Obese (yes vs normal/underweight)			2.1**[1.2,3.5]	2.1**[1.2,3.5]	2.1**[1.2,3.5]	2.1**[1.2,3.5]	2.1**[1.3,3.6]
Trying to lose weight (yes vs. no)			2.4***[1.6,3.7]	2.4***[1.6,3.7]	2.4***[1.6,3.7]	2.4***[1.6,3.7]	2.5***[1.6,3.9]
Victimization (yes vs. no)			1.6*[1.1,2.4]	1.6*[1.1,2.4]	1.6*[1.1,2.4]	1.6*[1.1,2.4]	1.6*[1.1,2.4]
Self-perception of unhappiness (Fully unhappy/not happy vs Fully happy/very happy/happy)			1.8**[1.2,2.8]	1.8*[1.1,2.7]	1.8*[1.1,2.7]	1.8**[1.1,2.7]	1.7*[1.1,2.7]
Cross-level interactions							
LGB prevalence in school (lower quartile) (level2)##LGB (yes vs. no) (level1)					1.2[0.3,4.8]		3.8[0.6,23.7]
LGB prevalence in school (lower quartile) (level2) ## Gender (female) (level1)						0.8[0.4,1.8]	1.0[0.4,2.2]
LGB prevalence in school (lower quartile) (level2)##LGB (yes vs. no) (level1))## Gender (female vs. male) (level1)							0.0[0.0,1.0]
LGB (yes vs. no) (level1))## Gender (female vs. male) (level1)							0.9 [0.3,2.8]
Contextual factors (School level)							
LGB prevalence in school (lower vs. middle-lower, middle-higher, and higher quartile)		1.0[0.8,1.4]		1.5*[1.0,2.2]	1.5[1.0,2.2]	1.7[1.0,3.0]	1.5[0.8,2.9]
Random part							
Area level variance (Random intercept)	0.2[0.1,0.4]	0.2[0.1,0.4]	0.2[0.0,2.1]	7.2e-1[0,.]	5.83e-06[0,.]	6.27e-06[0,.]	3.72e-07[0,.]
PCV&		-0%	−26.0%	−99.9%	−99.9%	−99.9%	−99.9%
Median Odds Ratio	MOR = 1.3	MOR = 1.3	MOR = 1.2	MOR = 1.0	MOR = 1.0	MOR = 1.0	MOR = 1.0
2 Res log-likelihood	2238.1	2238.1	755.8***	751.9***	751.8	751.6	746.1
Observations	2385	2385	883	883	883	883	883

* *p* < 0.05, ** *p* < 0.01, *** *p* < 0.001

## Discussion

Our findings did not support our initial hypothesis that we would find higher unhealthy weight-control behaviors among LGB youth attending public schools with lower LGB prevalence. Instead, we found that schools at middle-lower, middle-higher, and higher quartile of LGB prevalence protect all students, regardless of their sexual orientation.

At the individual level, LGB youth were significantly more likely to report unhealthy weight-control behaviors, which is in line with Lucassen et al. [31] Regarding the interaction between LGB x sex at the individual level, LGB boys were not significantly more likely to engage in unhealthy weight-control, although the direction of association was in the expected direction, in line with Hadland et al. [32]

However, the bigger picture from our study is that the lower LGB prevalence is correlated with unhealthy weight-control behaviors in *all* students. One reason for this is because heteronormal schools are more likely to transmit “toxic” gender norms for both heterosexual youth and LGB youth, which is consistent with previous studies [3, 4]. For heterosexual youth, these schools lead to unhealthy weight control behavior because boys & girls are pressured to conform to distorted ideal body types popularized by marketing & media (i.e. girls are expected to be thin & boys are expected to be trim) [33]. For LGB youth, the same schools are unhealthy because of bullying and minority stress. In other words, these schools are “lose-lose” for everyone.

Our findings suggest that addressing LGB prejudice in the Brazilians school context could promote positive health outcomes among adolescent students [34]. Other studies indicate that one of the first steps for creating a safe and supportive school climate is to reinstate sexual education in the curriculum [35]. Other examples of actions that improve school climate include presentation of LGB films [36], the presence of Gay-Straight Student Alliances in the school [37], and training for school staff to support sexual and gender minority students [4]. According to Gower et al. [4], schools that implemented more of the practices experienced lower victimization rates among their students, regardless of the sexual orientation of the students.

A limitation of our study is that the survey did not explicitly inquire about bullying specifically based on sexual orientation. Nor did we assess aspects of minority stress related to sexual orientation such as negative internalized stigma, discrimination related to sexual orientation, and acceptance by the family. Our survey also did not specifically inquire about sexual identity or attraction. The true LGB population was probably underestimated in our sample, since it was only defined by adolescents who reported having sexual intercourse with a same-sex partner, and did not count adolescents who were sexually inactive. In addition, our results may not be externally generalizable to other regions and areas in Brazil. Finally, we did not directly assess LGB-supportive climate in the schools; instead we used a proxy for LGB visibility evaluated by the prevalence of LGB students in schools.

## Conclusion

Our findings show that lower LGB prevalence in schools is associated with unhealthy weight-control behaviors regardless of sexual orientation. Further studies are needed to corroborate our findings, and improve on the limitations of our study, particularly the operationalization of school climate vis-a-vis sexual and gender minority students. In addition, the association may be due to residual confounding by other unobserved school characteristics, so that a more comprehensive assessment of school climate is warranted.

## Data Availability

The datasets used and analyzed during the current study are available from the corresponding author on reasonable request.

## Abbreviation

LGB: Lesbian, Gay, and Bisexual youth
